# Characterization of hyperglycemia due to sub-chronic administration of red ginseng extract via comparative global proteomic analysis

**DOI:** 10.1038/s41598-021-91664-8

**Published:** 2021-06-11

**Authors:** Ann-Yae Na, Jung Jae Jo, Oh Kwang Kwon, Piljoung Cho, Yan Gao, Ju-Hyun Kim, Kyu Min Kim, Sung Hwan Ki, Sangkyu Lee

**Affiliations:** 1 BK21 FOUR Community-Based Intelligent Novel Drug Discovery Education Unit, College of Pharmacy and Research Institute of Pharmaceutical Sciences, Kyungpook National University, Daegu, 41566 Republic of Korea; 2 College of Pharmacy, Yeungnam University, Gyeongsan, 38541 Republic of Korea; 3 College of Pharmacy, Chosun University, Gwangju, 61452 Republic of Korea

**Keywords:** Proteomics, Hepatology, Toxicology

## Abstract

Ginseng (*Panax ginseng* Meyer) is commonly used as an herbal remedy worldwide. Few studies have explored the possible physiological changes in the liver although patients often self-medicate with ginseng preparations, which may lead to exceeding the recommended dose for long-term administration. Here, we analyzed changes in the hepatic proteins of mouse livers using quantitative proteomics after sub-chronic administration of Korean red ginseng (KRG) extract (control group and 0.5, 1.0, and 2.0 g/kg KRG) using tandem mass tag (TMT) 6‐plex technology. The 1.0 and 2.0 g/kg KRG groups exhibited signs of liver injury, including increased levels of aspartate transaminase (AST) and alanine aminotransferase (ALT) in the serum. Furthermore, serum glucose levels were significantly higher following KRG administration compared with the control group. Based on the upregulated proteins found in the proteomic analysis, we found that increased cystathionine beta-synthase (CBS) and cystathionine gamma-lyase (CSE) levels promoted greater hydrogen sulfide (H_2_S) synthesis in the liver. This investigation provides novel evidence that sub-chronic administration of KRG can elevate H_2_S production by increasing protein expression of CBS and CSE in the liver.

## Introduction

Ginseng (*Panax ginseng* Mayer), is commonly been used as an herbal remedy in traditional medicine—it can be air-dried to produce white ginseng, or steamed or heated to produced red ginseng in order to preserve it and enhance its efficacy^1,2^. Korean red ginseng (KRG) and its components, such as ginsenosides, have been associated with various beneficial pharmacological effects on blood pressure, atherosclerosis, and hyperlipidemia by reducing oxidative damage^3–5^. Although KRG is considered safe for ingestion and tests on the safety of ginseng preparations have been conducted using animals, the US Food and Drug Administration has reported adverse effects arising from overexposure to functional foods including ginseng^6,7^. Recently, it has been reported that ginseng can damage vascular smooth muscle^8^ and lead to contractile dysfunction in vascular smooth muscle^9^; however, despite its long history and broad use, few studies have explored physiological changes in the liver after sub-chronic high-dose administration of ginseng.

The liver is a representative organ that detoxifies various absorbed xenobiotics, synthesizes proteins, and produces several biochemicals in mammalians for indispensable physiological functions. The liver, especially, is one of the main organs that supplies circulating blood and, consequently various tissues, with glucose^10^. Changes in blood glucose concentration may also occur depending on liver dysfunction. Furthermore, physiological changes in the liver are commonly caused by the use of exogenous compounds such as drugs, herbs, and alcohol^11^. These liver toxicities are well screened clinically with indicators such as aspartate transaminase (AST) and alanine aminotransferase (ALT)^12^, and the two indicators are used to directly judge the effect of ginseng on liver function^13^.

In this investigation, we show that significantly higher cystathionine beta-synthase (CBS) and cystathionine gamma-lyase (CSE) levels promote greater hydrogen sulfide (H_2_S) synthesis in the liver after sub-chronic administration of high-dose KRG. H_2_S is thought to be an endogenously produced gaseous signaling molecule, similar to nitric oxide (NO) and carbon monoxide, which plays a role in the regulation of inflammatory responses, apoptosis, oxidative stress, and angiogenesis^14–17^. Additionally, H_2_S is reportedly related to the regulation of glucose metabolism^18,19^. H_2_S is now recognized as an important cellular signaling molecule due to its important functions in several aspects of human health and disease^20^. Despite the beneficial effects of KRG in various physiological states, the way KRG affects H_2_S levels has not previously been explored. Considering the importance of the liver for H_2_S production, it is important to explore how H_2_S affects glucose metabolism and liver injury after long-term overexposed KRG administration. Thus, in this investigation, we explored the protein dynamics in the livers of mice after sub-chronic KRG administration using proteomic analysis.

## Results

### Changes in clinical characteristics

**Figure 1:**
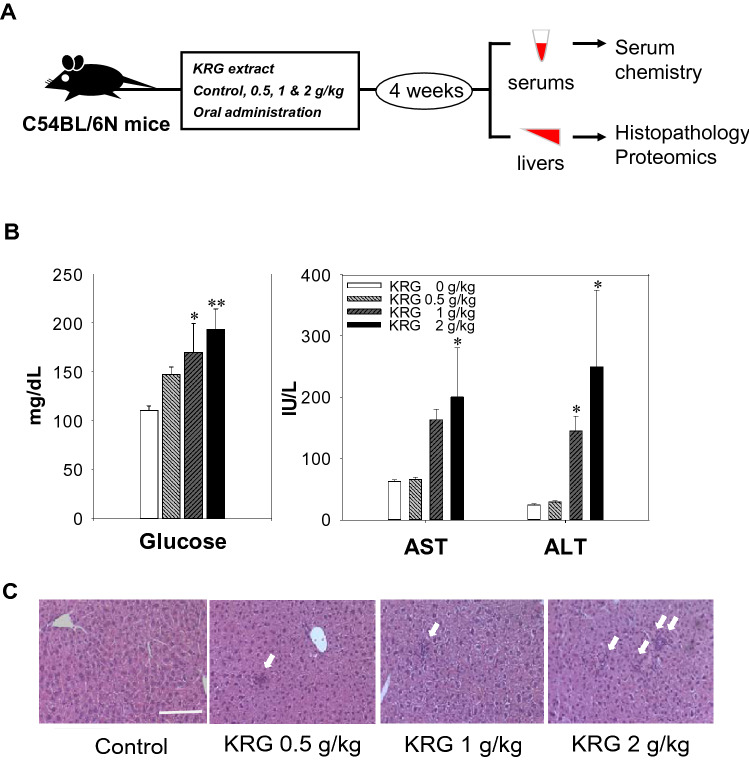
Overview of the experimental approach. Five-week-old C57BL/6 N male mice were orally administered different doses (0.5 g, 1.0 g, and 2.0 g/kg) of Korean red ginseng (KRG). After 4 weeks, serum and liver tissue were extracted for serum chemistry and proteomic analysis (**A**). Glucose levels and AST and ALT levels in the serum (**B**). Histological analysis of H & E staining of liver tissue at 200 × magnification. White arrows indicate inflammatory cell infiltration. Scale bar represents 150 mm. (**C**). Results are shown as the mean ± SEM of five to six animals per group. Statistical significance was set at * *p* < 0.05 and ** *p* < 0.01 compared with the control based on an ANOVA (SPSS Statistics 23 software package, https://www.ibm.com/analytics/spss-statistics-software).

In general, the daily recommended oral doses of ginseng for rodents is 500 mg/kg. These doses were calculated based on actual doses of red ginseng calculated for human beings (1.5–3.0 g/person/day)^21,22^. In this study, we administered KRG extract to mice orally every day for 28 days at a maximum of 4 times the recommended dose (0.5, 1.0, and 2.0 g/kg) to investigate the clinical effects of high-dose KRG (Fig. 1A). We quantified the ginsenoside contained in the KRG extract used in the experiment in our previous study^23^. These dosages have been used previously in general toxicity assessments in rodent models^24,25^. After 4 weeks of administration, we measured several serum parameters to characterize liver function. It was determined that glucose levels increased significantly, and serum AST and ALT levels were also significantly higher in the 1.0 and 2.0 g/kg KRG groups, respectively, which indicated liver injury (Fig. 1B). Furthermore, the hepatic pathologic change was confirmed in H&E stained tissues (Fig. 1C). Histopathological results were distinctly shown focal inflammatory cell infiltration in 2 g/kg KRG group compare to control group (White arrow). The liver function test and lipid profile results are summarized in Table S1. There were no other significant differences in terms of body weight, liver weight, or other serum parameters between groups.

### Global protein profiling in mice administered with KRG

**Figure 2:**
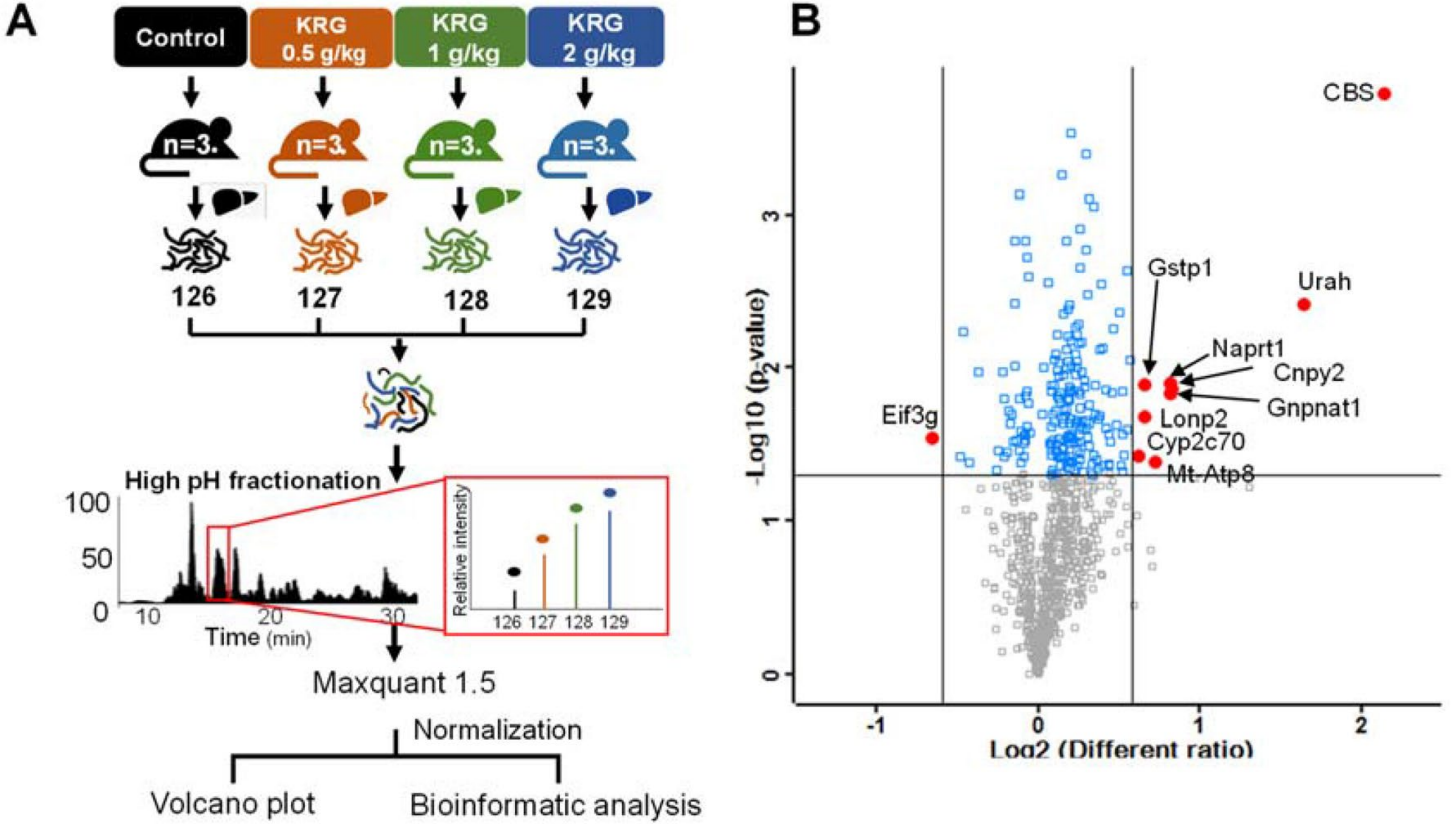
Overall scheme for the global proteomic profiling of mouse liver (**A**). Volcano plot and distribution of quantified proteins based on the reporter ion ratio in the three KRG groups (**B**).

The process used for protein profiling in this investigation is presented in Fig. 2A. In the 3 group, we identified a combined total of 1005 proteins, where 881 of them were quantified based on comparative proteomic analysis (Table S2). All data contained technical duplicates and the mass error for all identified peptides was assessed, with the highest mass error being > 0.02 Da (Fig. S1A). We conducted a Pearson correlation analysis of the reporter ion intensities to determine the quantitative accuracy of MS-based proteomics, with a resulting of 0.8775 (Fig. S1B).

### Classification of differentially expressed proteins after the administration of high-dose KRG

Proteins identified from the liver tissue of the 3 KRG groups were assigned a reporter ion ratio over the control group for tandem mass tag (TMT) analysis to identify the differentially expressed proteins (DEPs) associated with KRG-induced liver injury. We identified a total of 58 DEPs after comparing the 3 KRG groups and the control group (Table S3). A total of 19, 40, and 31 DEPs were identified at the 0.5, 1.0, and 2.0 g/kg KRG groups, respectively (log_2_ ratio ≥ 0.585 (upregulated) or log_2_ ratio ≤  − 0.585 (down-regulated)), for each KRG group over the control group (Fig. S1C). In addition, we employed one-way *t*-tests with a *p* value cutoff of 0.05 to identify proteins whose levels changed significantly after KRG administration. A Volcano Plot of DEP was illustrated by fold change (log_2_ Difference) versus significance (-Log10 *p* value) using a threshold value of 0.05. Protein IDs in red were considered significantly up- or down-regulated using the Perseus software package (Fig. 2B). Based on these criteria, 9 upregulated proteins showed statistical significance (Table 1).

**Table 1:** List of statistically significant upregulated proteins.

No	Accession	Protein name	Gene name	peptides	Protein coverage (%)	Ratio (KRG/control)			*p*-value
						0.5	1.0	2.0	
1	Q91WT9	Cystathionine beta-synthase	Cbs	3	3.9	4.48	4.24	4.54	0.000
2	Q9CRB3	5-hydroxyisourate hydrolase	Urah	2	13.6	2.73	3.21	3.48	0.004
3	Q8CC86	Nicotinate phosphoribosyltransferase	Naprt1	6	12.8	1.67	1.64	2.02	0.013
4	Q9JK38	Glucosamine 6-phosphate *N*-acetyltransferase	Gnpnat1	2	7.1	1.58	1.74	2.01	0.015
5	Q9QXT0	Protein canopy homolog 2	Cnpy2	2	14.3	1.55	1.88	1.92	0.014
6	Q7JCZ0	ATP synthase protein 8	mt-Atp8	2	34.3	1.39	2.00	1.65	0.040
7	P19157	Glutathione *S*-transferase P 1	Gstp1	8	41	1.42	1.70	1.63	0.014
8	Q9DBN5	Lon protease homolog 2, peroxisomal	Lonp2	2	2.6	1.54	1.80	1.42	0.022
9	Q91W64	Cytochrome P450 2C70	Cyp2c70	5	10.8	1.73	1.61	1.30	0.038

### Verification of cystathionine β-synthase (CBS) and cystathionine gamma-lyase (CSE) protein expression and H_2_S levels

**Figure 3:**
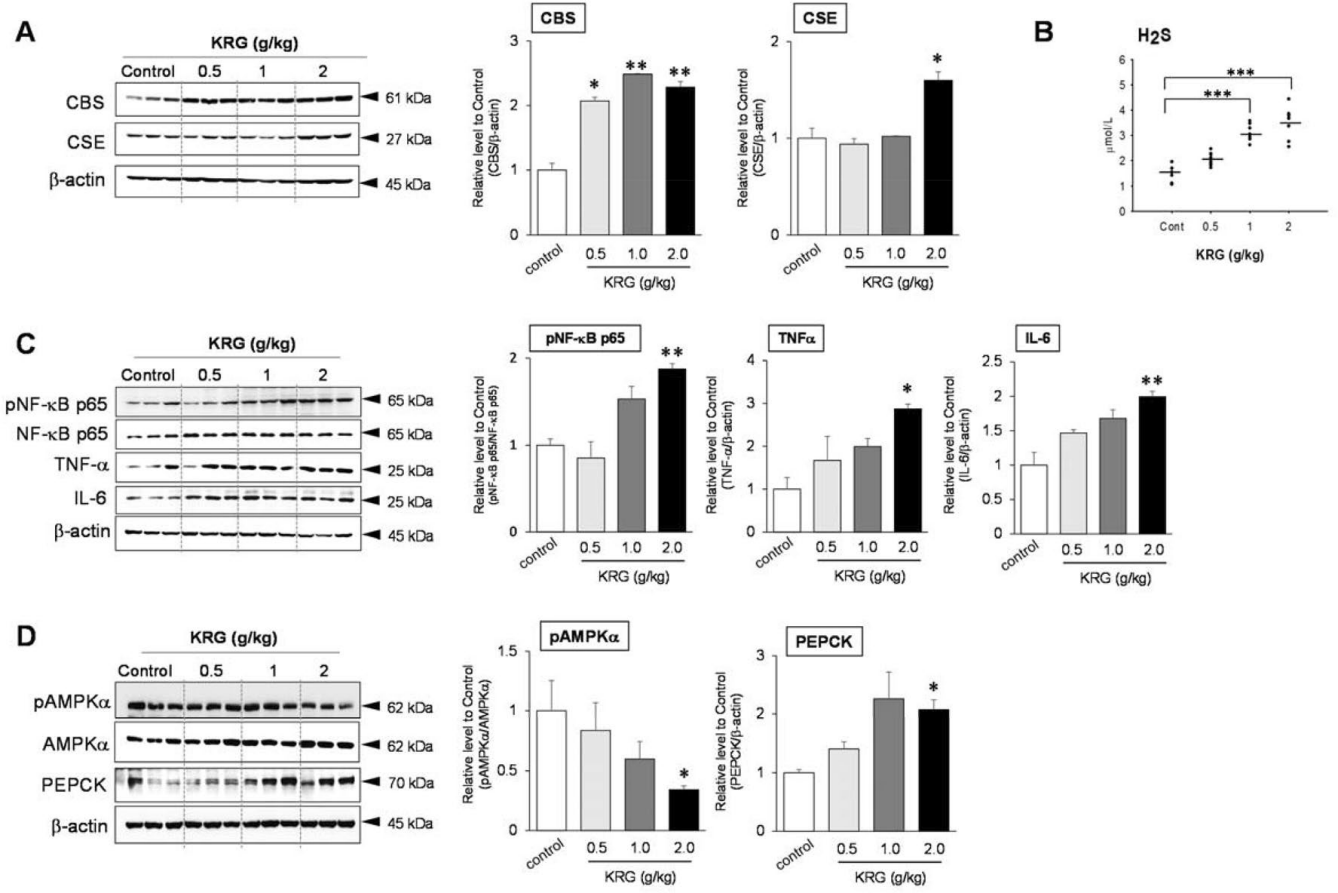
Verification of the changes in protein level in mouse liver following the administration of high-dose concentrations of KRG. Cystathionine β-synthase (CBS) and cystathionine γ-lyase (CSE) protein levels compared to β-actin via Western blots (**A**). H_2_S production based on ELISA (**B**). Changes in protein levels with increased H_2_S and the effects on liver injury (**C**) and the gluconeogenesis pathway (**D**) in mouse liver following the administration of high-dose concentrations of KRG. Bands were quantified using a densitometer in the ImageJ program, and the H_2_S results are expressed as mean ± SE (*n* = 5–6). Statistical significance was set at * *p* < 0.05, ** *p* < 0.01, and ***p < 0.001 compared with the control based on an ANOVA (SPSS Statistics 23 software package, https://www.ibm.com/analytics/spss-statistics-software).

After exploring the role of each protein and its association with hyperglycemia after KRG administration for 4 weeks, we focused on the most upregulated protein, CBS, and validated the accuracy of the TMT-labeled quantitative proteomics results using Western Blot (Fig. S2A). Furthermore, we determined that CSE is also an important protein in this study even though the proteomics results include no upregulation of CSE in the KRG groups. This is explained by the abundance of CBS and CSE in the liver and their involvement in the endogenous production of H_2_S and its metabolism^26^. CBS protein expression levels were found to be significantly higher in all KRG groups (0.5 g/kg; KRG relative level to control 2.07 (*p* < 0.05), 1 g/kg KRG; relative level to control 2.49 (*p* < 0.01), 2 g/kg KRG; relative level to control 2.28 (*p* < 0.01)), supporting the quantitative proteomics analysis. Also, the relative ratio of CSE protein expression was 1.6-fold (*p* < 0.01) which was increased in the only 2.0 g/kg KRG group (relative level to control, 1.0) (Fig. 3A). These changes in protein levels support the belief that dominant H_2_S-producing enzymes are highly expressed in the liver after high-dose KRG administration. When we measured the H_2_S levels in the liver samples using ELISA assays to further characterize CBS and CSE proteins, a significant dose-dependent increase in H_2_S concentration was seen in 1.0 and 2.0 g/kg KRG mouse liver tissues (3.1 umol/L, (*p* < 0.001); 3.5 umol/L, (*p* < 0.001)) (Fig. 3B).

### Protein expression levels and the association with oxidative stress and hyperglycemia through H_2_S

**Figure 4:**
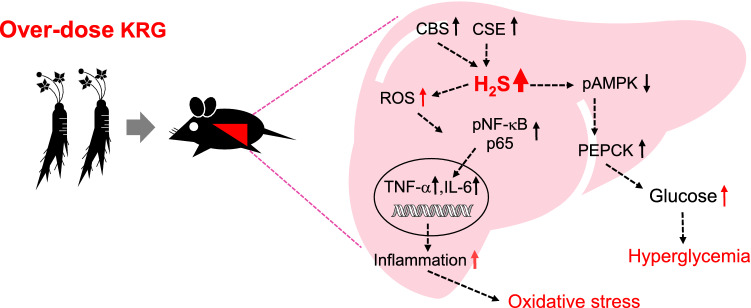
Signaling pathways underlying KRG-induced oxidative stress and hyperglycemia through H_2_S.

Phospho-NF-κB p65, NF-κB, TNF-α, and IL-6 expression levels were measured using Western Blot analysis to assess the inflammatory response to KRG over-dose (Fig. S2B). A significant increase in NF-κB p65 (relative level to control 1.88, (*p* < 0.01)), TNF-α (relative level to control 2.87, (*p* < 0.05)), and IL-6 (relative level to control 1.99, (*p* < 0.01)) was seen in the 2.0 g/kg KRG group compared to the control group (relative level to control 1.0) (Fig. 3C). Furthermore, to explore the role of H_2_S in the liver in terms of gluconeogenesis, we assessed the protein levels of phospho-5' AMP-activated protein kinase alpha (pAMPKα), AMPKα, and PEPCK, which are involved in the gluconeogenesis metabolic pathway (Fig. S2C). High levels of H_2_S activity in the liver dose-dependently inhibited the expression of phosphorylated AMPKα over AMPKα, with the 2.0 g/kg KRG group showing an almost 40% decrease in phospho-AMPKα/AMPKα protein levels (relative level to control 0.34, (*p* < 0.05)) (Fig. 3D). Interestingly, high-dose KRG increased PEPCK protein levels (2 g/kg KRG; relative level to control 2.81, (*p* < 0.05)) by increasing H_2_S levels. In summary, we demonstrated that KRG over-dose contributes to H_2_S synthesis, resulting in increased oxidative stress in the liver and hyperglycemia (Fig. 4).

### Gene ontology and Kyoto encyclopedia of genes and genomes pathway analysis of the DEPs based on bioinformatics

We analyzed the gene ontology (GO) and Kyoto Encyclopedia of Genes and Genomes (KEGG) pathways by calculating the Fisher’s exact test *p* value for 47 upregulated DEPs in order to increase our understanding of the mechanisms by which the sub-chronic administration of KRG in 3 groups, at different doses, led to liver injury. Fig. S3 summarizes the top five annotations from the Fisher exact tests for each category. The GO-based distribution of upregulated proteins was also evaluated to characterize the liver injury caused by sub-chronic high-dose KRG administration in mouse liver tissue. The KEGG pathway analysis showed that steroid hormone biosynthesis and sulfur metabolism were significantly enhanced with KRG administration. The domain annotation enrichment from the InterPro results indicated that cytochrome P450 was present at significantly higher concentrations in mouse liver after receiving high doses of KRG.

## Discussion

It has long been believed that ginseng is a nontoxic herbal medicine, but investigations have been conducted to determine its safety. Most of the previous ginseng research focused only on its protective effects as a medicinal herb for the treatment of a variety of medical conditions^27,28^. In this investigation, we showed for the first time that high levels of H_2_S may play an important role in regulating oxidative stress and hyperglycemia levels in the liver by sub-chronic high-dose KRG administration. To explore the mechanisms underlying the negative effects of high-dose KRG, an in vivo model was used for proteomic analysis with TMT labeling technology. We demonstrated that sub-chronic administration of high doses of KRG caused high serum AST and ALT levels and elevated blood glucose (Fig. 1B). Although we couldn’t find reference studies be comparable to subchronic administration of high dose KRG to normal mice, previous studies on liver function decline following long-term administration or high-dose administration for 90 days were confirmed. For examples, subchronic administration for 90 days of the main KRG metabolite of ginseng saponin, compound K, in beagle dogs showed increased ALT level in groups with compound K^29^. And a single case study reported KRG 6 g/day dose for 12 weeks, the liver function test showed increased AST and ALT level compared to prior liver enzymes (6 month earlier)^30^. These previous results could be suggest possibilities of negative effects on liver enzymes due to sub chronic high-dose KRG administration.

Although rare, as can be seen from the case of acute hepatotoxicity after consumption of ginseng-related products, the need for mechanistic research on hepatotoxicity derived from ginseng used as a health functional food is proposed. We listed 47 upregulated proteins to determine the biological mechanisms of KRG-induced hepatotoxicity. Interestingly, CBS was the most upregulated protein in all the KRG groups (Table 1) and H_2_S-generating reactions were catalyzed by CBS and CSE in the trans-sulfuration pathway^31^. Thus, the hyperglycemia caused by sub-chronic administration in the high-dose KRG groups may be associated with increased H_2_S synthesis via CBS and CSE in the liver, given that CSE is an important H_2_S-producing enzyme that can be upregulated by NO^26^. Several earlier studies that explored KRG treatment and its association with H_2_S showed, for example, that KRG treatment inhibited H_2_S in vivo and in vitro^32,33^. Choi et al. reported that KRG at doses of 50–100 μg/ml decreased both CBS and CSE expression in human umbilical vein endothelial cells (HUVECs)^34^. In this study, the protein expression levels of CBS and CSE determined by immunoblotting (Fig. 3a) were increased by administration of high-dose KRG. That difference is considered because the KRG dose within the recommended range for pharmacological effects significantly lowered the expression of inflammatory mediators.

An investigation of the process implicated in H_2_S-induced inflammation and ROS, determined that high H_2_S concentrations over a short period of time triggered the toxicity of H_2_S via the inhibition of mitochondrial cytochrome c oxidase and mitochondrial respiration^35^. Another study reported that administration of 500 μM NaHS could increase ROS formation through the inhibition of cytochrome c oxidase and the depletion of GSH in rat primary hepatocytes, which could lead to hepatotoxicity^36^. H_2_S exposure activated the NF-κB pathway, resulting in an increase in the protein levels of NF-κB, TNF-α, and IL-10. These results collectively indicate that H_2_S can induce oxidative stress via redox homeostasis disorders in the liver. Tan et al. (2017) reported H_2_S levels in wild-type mice liver tissue of approximately 1 μM/L^37^, which is similar to our results for the control group (1.43 μM/L) (Fig. 3b). Another study reported that 5-week HFD in mice induced a significant increase in hepatic H_2_S production, which was associated with elevated levels of CBS and CSE expression^31^.

H_2_S also reportedly stimulates gluconeogenesis and glycogenolysis, but inhibits glycogenesis and glycolysis, contributing to increased levels of glucose in the liver^18^. Overexpressed CSE in HepG2 cells stimulates H_2_S generation, resulting in attenuated glycogen storage. The signaling pathways for H_2_S are closely associated with gluconeogenesis and glucose production. These findings were supported by our immunoblotting results for glucose metabolism, including pAMPK and PEPCK (Fig. 3c). H_2_S activates PEPCK by strengthening the glucocorticoid receptors and blocking AMPK activity^18^ and stimulates G6Pase and FDP expression through key gluconeogenic transcription factor *S*-sulfhydrating PGC-1α^19^. Furthermore, other studies have reported that lower levels of H_2_S are related to liver dysfunction and result in hepatic fibrosis and cirrhosis, whereas higher levels of H_2_S strengthen insulin resistance and diabetes^38,39^.

In conclusion, we demonstrated that high H_2_S levels may be central to liver injury and elevated glucose levels may be due to sub-chronic administration of high-dose KRG (Fig. 4). This toxic mechanism is also associated with increased protein expression of CBS and CSE in the liver. In this study, KRG-induced liver toxicity was observed for the first time, and the results help increase our understanding of the biological mechanisms underlying KRG toxicity. Nonetheless, additional research under different physiological conditions is required to further delineate the mechanisms involved in ginseng toxicity.

## Methods

### Study design

The male C57BL/6 N mice were obtained from Orient Co. (Seongnam, Korea) and randomly housed at 4 mice per cage. The mice were acclimated for 1 week under controlled laboratory conditions (temperature of 22 ± 2 °C, humidity of 55 ± 5%, and 12-h light/dark cycle) before the experiments, and fed standard rodent chow and tap water ad libitum. The mice (mean body weight 20.3 ± 0.6 g) were randomly divided into 4 groups (6 mice per group) before administration of KRG. Daily oral administration of KRG in the treatment groups (0.5, 1.0, and 2.0 g/kg) took place over 4 weeks. The KRG extract used in this study was obtained from Punggi Ginseng Cooperative Association (Punggi, Korea) and was prepared using a traditional process that involves repeatedly steaming and drying the roots, hot-water extraction, and concentration^27^. The 13 ginsenosides Rb1, Rb2, Rc, Rd, Re, Rf, 20(S)-Rh1, 20(S)-Rh2, Rg1, 20(S)-Rg3, F1, F2, and compound K were absolutely quantified by LC–MS/MS^23^. The mice fasted for 12 h with free access to water before sacrifice to obtain blood and liver tissue. All animal experiments and methods were conducted in accordance with the guidelines of the Institutional Animal Care and Use Committee of Kyungpook National University and carried out in compliance with the ARRIVE guidelines.

### KRG preparation

We were provided with ginseng products by Punggi Ginseng Cooperative Association (Punggi, Korea). Thirteen ginsenosides were prepared according to a traditional routine process^40,41^. KRG is processed by placing the cleaned and sorted ginseng on bamboo or wooden shelves in a closed steam chamber and applying steam slowly. Steaming lasted approximately 50–90 min, depending on the size of the ginseng, until it was thoroughly prepared. Then the ginseng was transferred outdoors to cool and remove the moisture. Finally, the ginseng is moved to the baking room and baked at a low temperature until proper dryness is achieved.

### Serum chemistry

Blood samples were collected from the inferior vena cava and maintained at room temperature. The blood was centrifuged for 15 min at 4000 × *g* to obtain the serum samples. These samples were stored at − 80 ^o^C until they were tested. Serum parameters including liver function levels and lipid parameters were analyzed at the Hoseo Toxicological Research Center (Asan, Chungcheongnam-do, Korea) using a Hitachi 7020 Chemistry Analyzer (Hitachi, Tokyo, Japan).

### Liver sample preparation for proteomic analysis

Liver tissues (n = 3) of mice from each group were washed twice with cold phosphate-buffered saline and homogenized to extract the protein with 1% SDS buffer (1% SDS, 2 mM EDTA, 10 mM Tris–HCl, pH 7.5) containing a protease inhibitor cocktail (Thermo Fisher Scientific Inc., Rockford, IL). The liver homogenates were centrifuged at 12,000 × g for 10 min at 4 °C and the supernatants were transferred to new tubes. All samples from each group were pooled to minimize sample biological variation. Protein was reduced with 15 mM dithiothreitol and incubated at 56 °C for 30 min and then alkylated with 15 mM iodoacetamide at room temperature for 30 min in the dark. To purify the protein, 10% trichloroacetic acid was added to the protein samples and incubated for 4 h at 4 °C. The protein pellets were then washed twice with ice-cold acetone. The protein samples were solubilized in 100 mM triethylammonium bicarbonate buffer; then, Trypsin (Promega, Madison, WI) was added and the samples were incubated overnight at 37 °C. After trypsin digestion, the final concentration of 1% TFA was added to stop enzyme activation. The samples were then centrifuged at 12,000 × g for 10 min, the digested peptides were collected, and the 100 μg samples were obtained. The samples were kept in − 80 °C until use.

### Histopathology

Sliced liver samples (1 cm × 1 cm) taken from the largest lobe were fixed overnight with 10% formalin (Sigma Aldrich, St. Louis, US) in phosphate-buffered saline solution, embedded in paraffin, and stored at room temperature before analysis. A histological examination was performed at Histoire (Ansan, Gyeonggi-do, Korea) and Chosun University (Gwangju, Korea) to determine morphological changes. Liver tissues were stained with H&E and observed under 200X magnification.

### TMT labeling and high pH fractionation

A total of 40 µg of peptide from each group was labeled with 6-plex\TMT reagent (Thermo Fisher Scientific Inc., Rockford, lL), according to the manufacturer’s protocol. Briefly, the labels (Control, 126; KRG 0.5 g/kg, 127; KRG 1 g/kg, 128; KRG 2 g/kg, 129) were dissolved in 41 μL acetonitrile (ACN) prior to labeling, and 20 μL was added to each sample for incubation. After incubating for 1 h, 8 μL of 5% hydroxylamine was added to stop each reaction for 15 min. Then, the samples were combined with the dried labeled peptides using vacuum centrifugation. High pH fractionation (Thermo Fisher Scientific Inc., Rockford, IL) was performed with 8 different buffers, according to the manufacturer’s protocol in order to increase proteome coverage. Briefly, 8 different buffers of 0.1% trimethylamine solution, including 5, 7.5, 10, 12.5, 15, 17.5, 20, and 50% ACN were used to elute the TMT-labeled peptides. All fractioned samples were cleaned with C18 Ziptips (Millipore, Billerica, MA), according to the manufacturer’s protocol. All samples were prepared for duplicate LC–MS/MS analysis.

### LC–MS/MS analysis

The digested samples were analyzed using a LTQ Velos-Orbitrap Mass Spectrometer (Thermo Fisher Scientific, Waltham, MA), linked online to nanoLC. Eight fractions from TMT-labeled peptides (2 μg) were directly injected into a nanospray ionization source equipped with a hand-made reverse-phase analytical column (Proteo C12 4 μm beads, 90 Å pore size, phenomenex Torrance, CA, USA). The separation with nanoLC was performed using a flow rate of 300 nL/min. For data-dependent acquisition, a 60-min binary linear gradient was used. The gradients started with 3% solvent B (100% ACN with 0.1% FA) for 2 min, 3–21% solvent B for 50 min, and 21–90% solvent B for 3 min using Eksigent nanoLC (SCIX, Redwood city, CA). The LTQ velos orbitrap was run set at 2.0 kV electrospray source voltage and data-dependent MS/MS mode. For the MS settings, the MS scan (300–1800 m/z) was set to have a resolution of 60,000 and 1.0 × 10^6^ automated gain control (AGC). Normalized collision energy at 40% with HCD mode, 0.1 ms activation time, and first mass fixed at 100 m/z. This was followed by collision-induced dissociation MS/MS scans for up to the 10 most abundant ions, with a resolution of 7500, 1.0 × 10^6^ AGC and 100 ms maximum ion injection time.

### Identification of differentially expressed proteins

MaxQuant 1.5 integrated with the Andromeda search engine was used for searching MS/MS data^42^. Tandem mass spectra were analyzed against a total of 51,444 mouse sequences in the UniProtKB/Swiss-Prot database concatenated with the reverse decoy database and common contaminants. Trypsin was specified as a cleavage enzyme, allowing for two missed cleavages. Carbamidomethylation on cysteine was set as a fixed modification, whereas methionine oxidation and protein *N*-term acetylation were set as variable modifications. For protein quantification through TMT labeling, we calculated the ratio of reporter ions using the TMT 6-plex method at MaxQaunt 1.5. All other parameters in MaxQuant were set to default values. We excluded contamination to obtain a high-quality protein list. The differentially expressed peptides include peptides with FDR ≤ 0.01, score > 40, and absolute log2-fold-changes 0.58 (1.5-fold), which are calculated from the TMT reagent ion reporter ratio of different-dose KRG groups, compared to the control group. DEPs are defined as proteins with a fold change greater than 1.5 or less than 0.666 in terms of relative abundance.

### Bioinformatics

The list of GO analysis, KEGG^43^, and InterPro were classified according to the top 5 enrichment annotations among the upregulated proteins. The basis for calculating enrichment values for the mouse proteins was achieved using DAVID 6.7 online software (https://david.ncifcrf.gov/)^44,45^. The high enrichment was categorized by the EASE score and a modified Fisher’s exact test *p* value below 0.05. Visualizations, such as the volcano plot, for proteomics results were drawn using Perseus 1.6 (http://www.perseus-framework.org)^46^.

### Immunoblot analysis

To confirm the CSE and CBS protein expression levels, 10 μg protein samples lysed in RIPA buffer (25 mM Tris–HCl, pH 7.6, 150 mM NaCl, 1% NP-40, 1% sodium deoxycholate, 0.1% SDS; Thermo Scientific) were prepared from the livers taken from each group. The proteins were resolved on a 10% gel and transferred to a polyvinylidine difluoride membrane (Millipore, Bedford, MA, USA). The membrane was then incubated overnight with primary antibodies at 4 °C. Anti-CSE (MBS2015730) was obtained from My BioSource (San Diego, CA, USA), and anti-CBS (#14782), anti-TNF-α (#6945), anti-IL-6 (#12153), anti-phospho-NF-κB p65 (#3033), anti-NF-κB p65 (#4764), anti-phospho-AMPKα (#2535), anti-AMPKα (#2532), and anti-*β*-actin (#4967) were obtained from Cell Signaling Technology (Canvers, MA, USA). Anti-PEPCK was purchased from Santa Cruz (Dallas, TX, USA). The secondary antibodies were incubated for 1 h at room temperature. Anti-Rabbit (#7074) and anti-Mouse (#7076) were obtained from Cell signaling Technology (Canvers, MA, USA). Bands were detected using an enhanced chemiluminescence system (Amersham, Buckinghamshire, UK).

### H_***2***_S assays

A total of 20 mg of homogenated liver samples were centrifuged for 15 min at 1500 × *g* with cold PBS. The supernatant was then transferred into new tubes and assayed immediately or aliquoted and stored at − 80 °C. H_2_S (Kamiya biomedical company, Seattle, WA, USA) was measured as per the manufacturer’s protocols. Briefly, the samples and standards were incubated at 37 °C for 1 h after mixing with 50 μL of conjugate solution in antibody pre-coated plates. Substrate solutions were then reacted in the plate for 10–15 min after 5 washes with wash buffer. The stop solution was added and absorbance was measured at 450 nm. All samples were quantified and standardized to the same protein concentration using the BCA kit and calculated per mg of protein after performing the H_2_S assay. Standard curves were constructed by plotting the average OD to generate a four-parameter logistic curve-fit. The H_2_S activity levels of all samples were calculated by adjusting the standard curve and divided by protein concentration.

### Statistical analysisStatistical analysis

Statistical analyses were conducted using the SPSS Statistics 23 software package (IBM Corp., Armonk, NY). All data were expressed as mean ± standard error. For variance homogeneity, the data were analyzed using the *F*-value in Levene’s test. If it was not significant, the ANOVA test was conducted, followed by Bonferroni’s multiple comparisons test. Differences between groups were considered significant at *p* < 0.05 (*), *p* < 0.01 (**), and *p* < 0.001 (***).

### Ethical statement

All experimental protocols were approved by a KNU Industry Foundation for Safety Management Systems at Kyungpook National University (Approved No. KNU-2017-0089-1).

## Supplementary Information

Supplementary Information 1.

Supplementary Information 2.

Supplementary Information 3.

## Data Availability

The mass spectrometry proteomics data have been deposited to the ProteomeXchange Consortium via the PRIDE partner repository with the dataset identifier PXD023731.
