# Supplementary material for: Characterization of hyperglycemia due to sub-chronic administration of red ginseng extract via comparative global proteomic analysis
Source: Sci Rep. 2021 Jun 11;11:12374. doi: 10.1038/s41598-021-91664-8 (PMC8196207; doi:10.1038/s41598-021-91664-8)
Supplement: Supplementary file 1 — Supplementary Information 1. [file 41598_2021_91664_MOESM1_ESM.docx]

Supporting information

**Characterization of hyperglycemia due to sub-chronic administration of red ginseng extract *via* comparative global proteomic analysis**

Ann-Yae Na^1^, Jung Jae Jo^1^, Oh Kwang Kwon^1^, Piljoung ChoP^1^PP, Yan GaoPP^1^, Ju-Hyun Kim^2^, Kyu Min Kim^3^, Sung Hwan Ki^3,#^, Sangkyu Lee^1,^*

*^1^BK21 FOUR Community-Based Intelligent Novel Drug Discovery Education Unit, College of Pharmacy and Research Institute of Pharmaceutical Sciences, Kyungpook National University, Daegu 41566, Republic of Korea*

*^2^College of Pharmacy, Yeungnam University, Gyeongsan 38541, Republic of Korea*

*^3^College of Pharmacy, Chosun University, Gwangju 61452, Republic of Korea*

**Table of Contents**

**Supplemental Table S1**. Body weight, lipid parameters and liver function levels in mouse serum after 4-weeks with sub-chronic administration of high-dose KRG.

**Supplemental Table S2**. Identified protein information

**Supplemental Table S3**. Different expressed protein information

**Supplemental Fig S1.** MS data validation and subcellular location in proteomic analysis. Distribution of mass error of all the identified peptides (A) Pearson correlation coefficient of TMT reporter ion intensity in technically duplicated samples (B) Venn diagram of differentially expressed proteins among three KRG groups/control (C).

**Supplemental Fig S2.** Immunoblotting analysis of cystathionine β-synthase (CSB) and cystathionine γ-lyase (CSE) protein level by Western blot and β-actin protein levels (A) Immunoblotting analysis of the pNF-κB p65, NF-κB p65, TNF-α, and IL-6 protein levels (B) Immunoblotting analysis of pAMPKα and PEPCK protein levels (C).

**Supplemental Fig S3.** Functional enrichment of upregulated proteins in the KRG group as biological process, cellular component, and molecular function by GO (A) and KEGG pathway and protein domain (B) enrichment analysis of upregulated proteins in KRG groups.

**Supplemental Table S1.** Body weight, lipid parameters and liver function levels in mouse serum after 4-weeks with sub-chronic administration of high-dose KRG.

| **Lipid parameter** | **KRG extract (n=6)** | | | | | | | | | | | |
| --- | --- | --- | --- | --- | --- | --- | --- | --- | --- | --- | --- | --- |
|  | **KRG 0 g/kg** | | | **KRG 0.5 g/kg** | | | **KRG 1 g/kg** | | | **KRG 2 g/kg** | | |
| **Body weight (g)** | 24.1 | ± | 0.3 | 24.3 | ± | 0.2 | 23.6 | ± | 0.2 | 23.7 | ± | 0.4 |
| **Liver weight (g)** | 0.9 | ± | 0.1 | 1.0 | ± | 0.1 | 1.1 | ± | 0.1 | 0.9 | ± | 0.0 |
| **Total cholesterol**  **(mg/dl)** | 106.7 | ± | 6.2 | 106.7 | ± | 13.5 | 101.7 | ± | 16.3 | 104.3 | ± | 13.1 |
| **HDL-C (mg/dl)** | 32.1 | ± | 3.2 | 34.3 | ± | 3.5 | 31.3 | ± | 5.7 | 30.1 | ± | 3.6 |
| **LDL-C (mg/dl)** | 12.3 | ± | 0.9 | 12.6 | ± | 2.3 | 14.3 | ± | 4.7 | 13.0 | ± | 3.2 |
| **VLDL-C (mg/dl)** | 59.8 | ± | 5.7 | 59.8 | ± | 13.4 | 59.4 | ± | 10.2 | 58.2 | ± | 7.3 |
| **Cholesterol/LDL ratio** | 8.7 | ± | 0.7 | 8.8 | ± | 2.4 | 7.5 | ± | 1.7 | 8.2 | ± | 1.1 |
| **Triglyceride (mg/dl)** | 51.2 | ± | 6.4 | 53.3 | ± | 14.0 | 48.0 | ± | 18.4 | 42.5 | ± | 8.4 |

| **Liver function levels** | **KRG extract (n=6)** | | | | | | | | | | | |
| --- | --- | --- | --- | --- | --- | --- | --- | --- | --- | --- | --- | --- |
|  | **KRG 0 g/kg** | | | **KRG 0.5 g/kg** | | | **KRG 1 g/kg** | | | **KRG 2 g/kg** | | |
| **ALP (IU/L)** | 424 | ± | 23.7 | 446.8 | ± | 44.0 | 425 | ± | 57.6 | 406.5 | ± | 31 |
| **LDH (IU/L)** | 968.8 | ± | 86.7 | 1285 | ± | 185.9 | 1249 | ± | 269 | 1181.3 | ± | 272.6 |
| **Total protein (g/dl)** | 5.9 | ± | 0.4 | 6.2 | ± | 0.4 | 5.9 | ± | 0.8 | 6.1 | ± | 0.3 |
| **Albumin (g/dl)** | 2.1 | ± | 0.2 | 2.1 | ± | 0.2 | 2 | ± | 0.3 | 2.1 | ± | 0.1 |
| **Albumin/globulin ratio** | 0.5 | ± | 0.0 | 0.5 | ± | 0.0 | 0.5 | ± | 0.1 | 0.5 | ± | 0.0 |
| **Total bilirubin (mg/dl)** | 0.05 | ± | 0.0 | 0.05 | ± | 0.0 | 0.05 | ± | 0.0 | 0.05 | ± | 0.0 |

**Supplemental Fig S1.**

**Supplemental Fig S2.**

**Supplemental Fig S3**
